# Anti-Seizure Medication Treatment of Benign Childhood Epilepsy With Centrotemporal Spikes: A Systematic Review and Meta-analysis

**DOI:** 10.3389/fphar.2022.821639

**Published:** 2022-03-10

**Authors:** Wenwen Cheng, Yan Yang, Ying Chen, Sharui Shan, Changhui Li, Ling Fang, Weiguo Zhang, Song Lan, Xiong Zhang

**Affiliations:** ^1^ Department of Neurology, Maoming People’s Hospital, Maoming, China; ^2^ Department of Ideological and Political Theory Teaching, Maoming Polytechnic, Maoming, China; ^3^ Department of Rehabilitation, The First Affiliated Hospital of Guangdong Pharmaceutical University, Guangzhou, China

**Keywords:** BECTS, efficacy, tolerability, meta-analysis, anti-seizure medication (ASM)

## Abstract

**Background:** This study aimed to evaluate the efficacy and tolerability of Anti-Seizure medication (ASM) treatment in patients with BECTS.

**Method:** We searched PubMed, Cochrane Library, Embase, MEDLINE, Web of Science, China National Knowledge Infrastructure (CNKI), WANFANG DATA, and China Science and Technology Journal Database (VIP) between 1 Jan 1990, and 1 Sep 2021, for randomized controlled studies. Data on seizure freedom rate, rate of treatment withdrawal due to serious adverse events, rate of any adverse events and dropout, 50% remission rate, the proportion of patients whose EEG to be normalized, and improvement in cognitive function were extracted by two authors independently. The pooled data were meta-analyzed using a random effects model.

**Results:** A total of 27 studies evaluating 9 ASMs were included, 19 of which were suitable for meta-analysis. Compared with sulthiame (STM), levetiracetam (LEV) was associated with a higher probability of treatment withdrawal due to serious adverse events [RR = 5.12, 95% CI (1.19, 22.01), *I* ^*2*^ = 0.0%], experiencing any adverse events [RR = 5.12, 95% CI (1.19, 22.01)], and dropping out for any reason [RR = 3.17, 95% CI (1.36, 10.11)], while it did not affect the seizure freedom rate [RR = 0.90, 95% CI (0.75, 1.06)]. LEV significantly improved cognitive performance relative to carbamazepine (CBZ) but had no effect on the proportion of any adverse events [RR = 0.62, 95% CI (0.25, 1.59)] and EEG to be normalized [RR = 1.27, 95% CI (0.94, 1.71)]. There was no higher probability of a 50% remission rate when comparing valproic acid (VPA) to LEV [RR = 0.96, 95% CI (0.57, 1.61)] and oxcarbazepine (OXC) [RR = 0.61, 95% CI (0.31, 1.20)]. In addition, STM was related to a higher probability of EEG normalization than placebo [RR = 4.61, 95% CI (2.12, 10.01)]. The included single studies also provided some evidence for the efficacy and/or tolerability of other ASMs in BECTS, including topiramate, lamotrigine, clobazam, and clonazepam. The risk of bias of the included studies was frequently low or unclear.

**Conclusion:** This study indicated some discrepancies in efficacy and tolerability among ASMs used in patients with BECTS. More randomized controlled trials (RCTs) comparing ASMs with larger populations are required to ascertain the optimum antiepileptic drug treatment to guide clinicians.

## Key Points

• Sulthiame is better tolerated than levetiracetam but without a difference in efficacy
• Levetiracetam demonstrated comparable safety to carbamazepine but better cognitive improvement
• Levetiracetam is not superior to valproic acid in efficacy and tolerability
• Oxcarbazepine was not better at seizure control and EEG normalization than valproic acid

## 1 Introduction

Benign childhood epilepsy with centrotemporal spikes (BECTS), also known as Rolandic epilepsy, is the most common focal epilepsy syndrome in children, accounting for approximately 13–23% of the total number of children with epilepsy (Shields and Snead, 2009). The age of onset ranges from 3 to 13 years with a peak at 9–10 years, and in children under 13 years of age, the incidence is approximately 10–20 per 100,000 (Lüders et al., 1987; Holmes, 1993). In general, BECTS is considered a “benign” disease with a favorable prognosis because of its infrequent clinical seizure and spontaneous remission in adolescence. However, a larger number of studies in recent years have shown that children with BECT have extensive neuropsychological impairment involving language ability, memory, emotion, motor function, and many other aspects (Garcia-Ramos et al., 2015; Kirby et al., 2017; Wickens, Bowden and D'Souza, 2017), and cognitive impairment seems to correlate with epileptic activity on electroencephalogram (EEG) (Massa et al., 2001; Xiao et al., 2016). Based on this, the rational use of ASMs is necessary to control seizures and improve neuropsychological dysfunction and/or EEG changes in children with BECTS.

However, the treatment of patients with BECTS is controversial, and the risks versus benefits of using ASMs are the core of the treatment controversy. ASMs that can effectively control seizures and cause fewer adverse reactions are preferred for the treatment of BECTS. To date, there is no uniform standard for the clinical application of ASMs treated in BECTS, and regional differences in the ASMs of choice exist. For example, levetiracetam (LEV) is used as the first-line treatment for BECTS patients in the United States, sulthiame (STM) is preferred in Germany, Austria, Israel, Japan and other countries, and valproate acid (VPA) is used in France. In China, oxcarbazepine (OXC), VPA and LEV are the three most commonly used ASMs in the treatment of BECTS (Liu et al., 2017; Gu et al., 2020). Moreover, the efficacy and safety of different ASMs are unclear. VPA is one of the most classical ASMs; it has efficacy in seizure control and was recommended for the treatment of BECTS by the International League Against Epilepsy 2017 (Bhasin and Sharma, 2019). A study conducted by Xiao et al. also demonstrated that low-dosage VPA exhibited better efficacy than LEV in improving the electrophysiological abnormalities of children with BECTS (Xiao et al., 2014). Two studies performed by Wang et al. and Sun et al. applied the Wechsler Intelligence Scale to explore the effect of ASMs and revealed that VPA was associated with a worse effect on cognitive improvement than OXC and LEV (Sun, 2015; Wang WX et al., 2019). Similar discrepant results of other ASMs can also be seen in different comparative studies (Kanemura et al., 2018; Kim et al., 2018; Tacke et al., 2018).

Therefore, evaluating the efficacy and safety of anti-seizure medication for BECTS patients is of great guiding significance for clinical medication. Two published articles have reviewed and summarized this issue and reported using STM, LEV, or clobazam as first-line agents for the treatment of BECTS (Tan et al.,2014; Gerstl et al., 2021). However, one of the two reviews included only four RCT studies because of its early publication date and the language limitation of publication (Tan, et al., 2014), and the result of the other review only examined the seizure freedom rates as efficacious with fewer databases (Gerstl, et al., 2021). Hence, the results of these published reviews might be inadequate and inaccurate, and the effects of ASM therapy in BECTS patients remain unclear.

In this study, we performed a systematic review and meta-analysis of all available randomized control trials (RCTs) to comprehensively evaluate the efficacy and tolerability of the use of ASMs in patients with BECTS. Only RCTs were included, and quantitative synthesis (meta-analysis) was only performed when at least two studies were available for each outcome.

## 2 Methods

The present study was conducted based on the Preferred Reporting Items for Systematic Reviews and Meta-Analyses (PRISMA) guidelines and the Cochrane Handbook for Systematic Reviews of Interventions (Moher et al., 2015; Cumpston et al., 2019). The final protocol was registered with PROSPERO (www.crd.york.ac.uk/PROSPERO/, number CRD42021276942).

### Search Strategy

The electronic databases PubMed, Cochrane Library, Embase, MEDLINE, Web of Science, China National Knowledge Infrastructure (CNKI), WANFANG DATA, and China Science and Technology Journal Database (VIP) were searched for studies. The search terms were “Epilepsy, Rolandic OR Rolandic Epilepsy OR Benign Rolandic Epilepsy of Childhood OR Benign Epilepsy With Centrotemporal Spikes OR BECTS AND Antiepileptic drugs OR Anticonvulsants OR Valproic acid OR Carbamazepine OR Lamotrigine OR Oxcarbazepine OR Levetiracetam OR Topiramate OR Perampanel OR Sulthiame OR Gabapentin OR Clonazepam AND randomized controlled trial OR randomized OR placebo.” The database search was run from 1 Jan 1990, to 1 Sep 2021. We also hand-searched the reference lists of articles that were considered for inclusion in the review.

### Study Selection and Outcomes

Two reviewers (YY and YC) first independently evaluated the literature to select the studies based on the title and abstract. The full text of an article was obtained when either reviewer considered that it might fulfill the inclusion criteria and then evaluated the selected full-text articles for inclusion in this systematic review. Studies were included according to the following inclusion criteria: 1) patients with a diagnosis of BECTS; 2) patients treated with ASMs (as monotherapy or in polytherapy); 3) all randomized controlled trials (RCTs) that compared the use of different ASMs or compared the use of ASMs with placebo, or both; 4) no restriction on country, sex, setting, or language of publication; and 5) failure to report the specific duration of drug treatment and observation will be excluded as low literature quality.

The primary outcomes of this study included the proportion of patients who achieved seizure remission (i.e., seizure freedom) throughout an observation period after randomization, the proportion of patients who experienced serious adverse events leading to treatment discontinued or changed (treatment withdrawal). The secondary outcomes included the proportion of patients who experienced any adverse events, the proportion of patients who dropped out for any reason, the proportion of patients with a reduction in seizure frequency of more than 50% compared to baseline (called 50% remission rate below), the proportion of patients whose EEG to be normalized, and improvement in cognitive function.

### Data Collection and Extraction

Data were independently extracted by two authors (SR-S and CH-L) using a standardized data-recording form, and disagreements between authors were discussed with the corresponding author. The following information was extracted from the included studies: 1) study characteristics: first author, year of publication, study country, sample size, and language of publication; 2) population characteristics: mean/median age and sex; 3) ASM treatment: ASM type, dosage, and duration; and 4) outcomes: primary and secondary outcomes. A Java program called Plot Digitizer (http://plotdigitizer.sourceforge.net/) was applied to convert plotted values into numerical form if adequate information was not provided by the study. The corresponding authors or the first authors were contacted if there were missing data.

### Quality Assessment

Each included study was assessed by two reviewers (LF and WG-Z) independently. The risk of bias in the literature was assessed by The Cochrane Collaboration’s tool across seven domains: random sequence generation, allocation concealment, blinding of participants and personnel, blinding of outcome assessment, missing data, selective reporting, and other biases (Cumpston, et al., 2019). The risk of bias for each quality criterion was reported as high, low, or unclear. Any disagreements on the risk of bias assessment were resolved by consensus.

### Data Synthesis and Statistical Analysis

All data analyses were performed using STATA 13.0 software. Meta-analyses were only performed when at least two studies were available for each outcome. We calculated the effect size by risk ratios (RRs) for dichotomous data and weighted mean differences (WMDs) for continuous data with 95% confidence intervals. A random effects model was chosen to synthesize the effect sizes. The heterogeneity across each effect size was evaluated with *I* ^*2*^ statistics, and we considered an *I* ^*2*^ > 75, 50, and 25% as notable, moderate, and mild heterogeneity, respectively. When there was notable heterogeneity for an intervention comparison, only a narrative synthesis of the results was presented. Subgroup analysis was planned according to the age of the participants (>12 and <12). Since no more than 3 studies were included in the meta-analysis of most outcomes, we did not perform the proposed subgroup analysis in this study.

According to the Cochrane Handbook for Systematic Reviews of Interventions, at least 10 studies are needed to identify opportunity asymmetry due to publication bias in funnel plots (Cumpston, et al., 2019). None of our intervention comparisons included an inadequate number of studies to meet this prerequisite; hence, publication bias was not estimated in this review.

## 3 Results

**FIGURE 1:**
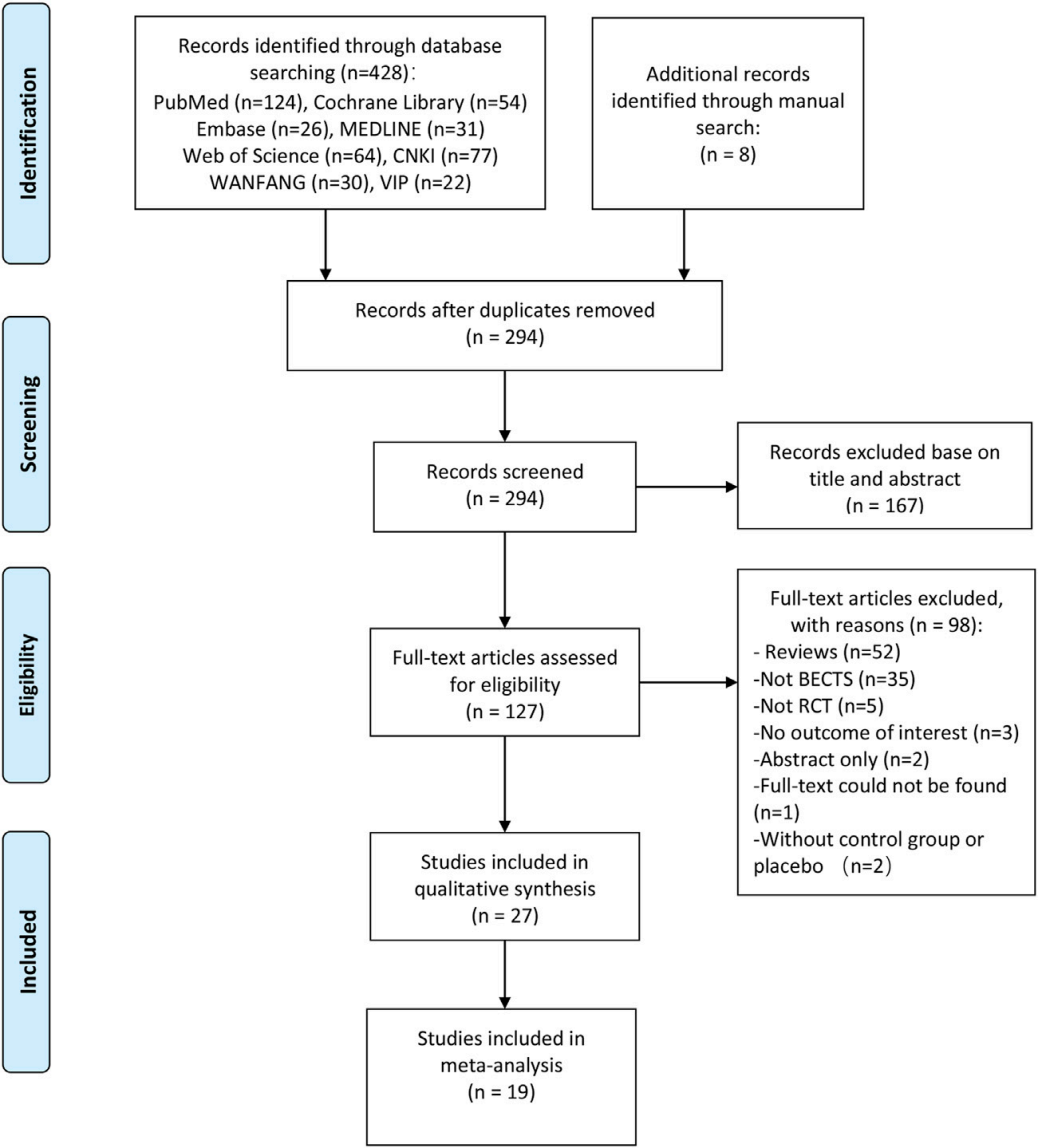
Flow diagram of the study selection and inclusion process.

The initial literature search identified 436 citations. After removing duplicate articles and screening titles and abstracts, 47 articles were selected for a full-text review. According to our eligibility criteria, 27 articles with 1737 participants were included in our systematic review. Of the 27 records included (Mitsudome et al., 1997; Rating et al., 2000; Bast et al., 2003; Coppola et al., 2007; Kang et al., 2007; Andrade et al., 2009; Li SM et al., 2009; Li ZH and Huang, 2011; Zhou T, 2011; Gu HF, 2012; Tao and Wang, 2012; Borggraefe et al., 2013; Kwon et al., 2013; CH, 2014; Sun, 2015; Zhang J et al., 2015; Chen CY and Zheng, 2016; JY, 2016; Li J, 2016; Tacke et al., 2016; NS, 2017; Tacke, et al., 2018; Ahadi et al., 2020; Zhang et al., 2020; Du ZZ et al., 2021; LY, 2021; Suo et al., 2021), 19 studies involving 1,224 subjects were suitable for meta-analysis (Mitsudome, et al., 1997; Rating, et al., 2000; Bast, et al., 2003; Coppola, et al., 2007; Li SM et al., 2009; Li ZH and Huang, 2011; Zhou T, 2011; Gu HF, 2012; Tao and Wang, 2012; Borggraefe, et al., 2013; CH, 2014; Sun, 2015; Chen CY and Zheng, 2016; JY, 2016; Li J, 2016; Tacke, et al., 2016; Ahadi, et al., 2020; Suo, et al., 2021; Zhang et al., 2020). A summary of the literature search according to the PRISMA flowchart is presented in Figure 1.

### Characteristics of the Included Studies

This study included 27 studies for qualitative synthesis. One trial conducted from the same pool of participants led to two publications, which were counted as two separate studies in our meta-analysis (Tacke, et al., 2016; Tacke, et al., 2018). The total sample consisted of 1,188 children with BECTS and 1,074 control participants. Fifteen of these 27 studies were published in Chinese (Mitsudome, et al., 1997; Rating, et al., 2000; Li SM et al., 2009; Li ZH and Huang, 2011; Zhou T, 2011; Gu HF, 2012; Tao and Wang, 2012; CH, 2014; Sun, 2015; Zhang J et al., 2015; Chen CY and Zheng, 2016; JY, 2016; Li J, 2016; NS, 2017; Zhang et al., 2020; Du ZZ et al., 2021; LY, 2021), 11 in English (Bast, et al., 2003; Coppola, et al., 2007; Kang, et al., 2007; Borggraefe, et al., 2013; Kwon, et al., 2013; Tacke, et al., 2016; Tacke, et al., 2018; Ahadi, et al., 2020; Suo, et al., 2021), and one in Spanish (Andrade, et al., 2009). Five studies compared the use of ASMs with placebo (Rating, et al., 2000; Bast, et al., 2003; Kwon, et al., 2013; NS, 2017; LY, 2021), and the remaining 21 studies compared the efficacy of different ASMs (Mitsudome, et al., 1997; Coppola, et al., 2007; Kang, et al., 2007; Andrade, et al., 2009; Li SM et al., 2009; Li ZH and Huang, 2011; Zhou T, 2011; Gu HF, 2012; Tao and Wang, 2012; Borggraefe, et al., 2013; CH, 2014; Zhang J et al., 2015; Sun, 2015; Chen CY and Zheng, 2016; Li J, 2016; JY, 2016; Tacke, et al., 2016; Tacke, et al., 2018; Ahadi, et al., 2020; Zhang et al., 2020; Du ZZ et al., 2021; Suo, et al., 2021). The eligible studies examined the effect of ASMs, including LEV, CBZ, STM, TPM, OXC, VPA, LTG, CLB, and CZP. The characteristics of the 27 included studies are presented in Table 1. Moreover, patients belong to different healthcare system enjoy different levels of medical services (Meng et al., 2019), it was necessary to highlight the level of healthcare delivery provided to patients that enrolled in eligible studies. Finally, none of the studies included in this paper reported the level of healthcare delivery that patients received.

**TABLE 1:** Summary of included study characteristics.

Study	Country	Language of publication	Number of patients	Mean Age, Y (range)	Sex (male%)	Treatment (participants, n)	Primary and secondary outcomes
Ahadi et al. (2020)	Iran	English	94 randomized, 92 analyzed	LEV 8.7 ± 2.766, CBZ 8.36 ± 2.250	LEV 56.5%, CBZ 60.9%	LEV: initial dose of 25–30 mg/kg/day (46), CBZ: the initial dose of 15–20 mg/kg/day (46)	Primary: seizure freedom rate, treatment withdrawal due to any serious adverse events. Secondary: any adverse events, rate of dropped out
Andrade et al. (2009)	Spain	Spanish	43 randomized	CLB 9.22 ± 1.3, CBZ 8.4 ± 1.3	CLB 66.7%, CBZ 40%	CLB: initial dose of 1 mg/kg/day, maximum of 5 mg/kg/day, (18). CBZ: initial does 10 mg/kg/day, maximum of 30 mg/kg/day, (25)	Primary: effects on seizure freedom, treatment withdrawal due to any serious adverse events. Secondary: any adverse events, effects on cognition
Bast et al. (2003)	Europe	English	66 randomized	Mean 8.3 for total (3.1–10.7)	60.7% for total	STM: 5 mg/kg/day (31)	Primary: the rate of treatment failure events (TFEs). Secondary: the proportion of patients whose EEG to be normalized
						Placebo (35)	
Borggraefe et al. (2013)	Germany	English	47 randomized, 43 analyzed	LEV 8.7 ± 1.7, STM 9.0 ± 1.5	LEV 81.4%, STM 54.5%	LEV: started in a dosage of 10 mg/kg, increments of 10 mg/kg, a final dosage of 30 mg/kg (21)	Primary: seizure freedom rate, treatment withdrawal due to any serious adverse events. Secondary: any adverse events, rate of dropped out
						STM: started in a dosage of 2 mg/kg, increments of 2 mg/kg, a final dosage of 6 mg/kg (22)	
Chen CY and Zheng, (2016)	China	Chinese	150 randomized	8.2 ± 1.7 for total (2–12)	58.0% for total	OXC: started in a dosage of 5–10 mg/kg, increments of 5–10 mg/kg, a final dosage of 30–40 mg/kg (75)	Primary: seizure freedom rate. Secondary: 50% remission rate
						VPA: started in a dosage of 10 mg/kg, increments of 5–10 mg/kg, a final dosage of 20–40 mg/kg (75)	
Coppola et al. (2007)	Italy	English	39 randomized	LEV 10.5, OXC 8.4	LEV 52.4%, OXC 55.6%	LEV: target dose 20 mg/kg/day, maximum 30 mg/kg/day (21)	Primary: seizure freedom rate, treatment withdrawal due to any serious adverse events. Secondary: any adverse events
						OXC: target dose 20 mg/kg/day, maximum 35 mg/kg/day (18)	
Du ZZ et al. (2021)	China	Chinese	60 randomized	LTG 8.62 ± 1.12, LEV 8.72 ± 1.23	LTG 54.8%, LEV 51.7%	LTG: started in a dosage of 0.3 mg/kg/day, increments of 0.5 mg/kg/day, a final dosage of 2–5 mg/kg/day (31). LEV: started in a dosage of 10 mg/kg/day, increments of 10 mg/kg/day, a final dosage of 30–40 mg/kg/day (29)	Secondary: any adverse events
Gao et al., 2014	China	Chinese	47 randomized, 69 analyzed	VPA 8.40 ± 2.45, CBZ 8.65 ± 2.36	VPA 57.1%, CBZ 55.9%	VPA: started in a dosage of 10 mg/kg/day, a final dosage of 20–30 mg/kg/day (35)	Primary: seizure freedom rate. Secondary: 50% remission rate, the proportion of patients whose EEG to be normalized
						CBZ: started in a dosage of 8–10 mg/kg/day, maximum dosage of 20–30 mg/kg/day (34)	
Gu, 2012	China	Chinese	80 randomized	8.21 for total (6–12)	47.5% for total	LEV: started in a dosage of 10 mg/kg/day, increments of 10 mg/kg/day, maximum dosage of 20 mg/kg/day (40)	Secondary: the proportion of patients whose EEG to be normalized, improvement in cognitive function
						CBZ: 10–20 mg/kg/day (40)	
Kang et al. (2007)	Korea	English	112 randomized	TPM 8.7, CBZ 8.7	TPM 55.2%, CBZ 59.3%	TPM: minimum dose 50–75 mg/day, maximum 4 mg/kg/day (58)	Primary: seizure freedom rate, treatment withdrawal due to any serious adverse events. Secondary: any adverse events, rate of dropped out, improvement in cognitive function
						CBZ: minimum dose 20 mg/kg/day, maximum 30 mg/kg/day (54)	
Kwon et al. (2013)	Korea	English	39 randomized, 29 analyzed	OXC 8.2 ± 2.3, placebo 8.5 ± 2.3	OXC 46.2%, placebo 68.8%	OXC: started in a dosage of 5–10 mg/kg/day, increments of 10–20 mg/kg/day (13)	Primary: seizure freedom rate. Secondary: rate of dropped out and 50% remission, the proportion of patients whose EEG to be normalized, improvement in cognitive function
						Placebo (16)	
Li SM et al. (2009)	China	Chinese	80 randomized	9.12 for total (6–12)	57.5% for total	LEV: started in a dosage of 10 mg/kg/day, increments of 10 mg/kg/day, maximum dosage of 20 mg/kg/day (40)	Secondary: improvement in cognitive function
						CBZ: 10–20 mg/kg/day (40)	
Li ZH and Huang, (2011)	China	Chinese	80 randomized	(4–10) for total	60% for total	LEV: started in a dosage of 10 mg/kg/day, increments of 10 mg/kg/day, maximum dosage of 20 mg/kg/day (15)	Primary: seizure freedom rate. Secondary: 50% remission rate, any adverse events
						VPA: 20 mg/kg/day (15)	
Li J, (2016)	China	Chinese	80 randomized	OXC 7.1 (4–10), VPA 6.3 (2–10)	OXC 63.3%, VPA 60%	OXC: started in a dosage of 10 mg/kg/day, increments of 10 mg/kg/day, maximum dosage of 20–30 mg/kg/day (30)	Primary: seizure freedom rate. Secondary: 50% remission rate, any adverse events, the proportion of patients whose EEG to be normalized
						VPA: started in a dosage of 10 mg/kg/day, increments of 5 mg/kg/day, maximum dosage of 20–30 mg/kg/day (30)	
Mitsudome et al. (1997)	Japan	English	80 randomized	CZP 7.3 (3.11–9.11), VPA 8.6 (4.0–10.11), CBZ 8.6 (5.5–10.3)	CZP 55.0%	CZP: 0.015–0.04 mg/kg/day (20)	Secondary: the proportion of patients whose EEG to be normalized
					VPA 60.0%	VPA: 13–18 mg/kg/day (10)	
					CBZ 50.0%	CBZ: 3.1–6.5 mg/kg/day (10)	
Rating et al. (2000)	Europe	English	66 randomized	STM 8.2 (3.9–10.7), placebo 8.4 (3.1–10.3)	STM 51.6%, placebo 68.6%	STM: 5 mg/kg/day (31)	Primary: seizure freedom rate, treatment withdrawal due to any serious adverse events. Secondary: rate of dropped out, the proportion of patients whose EEG to be normalized
						Placebo (35)	
Su et al., 2017	China	Chinese	48 randomized	LEV + VPA 4.29 ± 0.38, VPA 4.31 ± 0.40	LEV + VPA 66.7%, VPA 70.8%	VPA: started in a dosage of 5 mg/kg/day, increments of 2.5 mg/kg/day, maximum dosage of 50–60 mg/kg/day (24)	Secondary: any adverse events, improvement in cognitive function
						LEV: started in a dosage of 10 mg/kg/day, increments of 10 mg/kg/day, maximum dosage of 20 mg/kg/day (24)	
Sun, (2015)	China	Chinese	38 randomized	LEV 8.35 ± 2.12, VPA 8.45 ± 2.04	LEV 68.4%	LEV: started in a dosage of 20 mg/kg/day, increments of 10 mg/kg/day, maximum dosage of 30–40 mg/kg/day (19)	Primary: seizure freedom rate. Secondary: 50% remission rate, any adverse events, the proportion of patients whose EEG to be normalized, improvement in cognitive function
					VPA 57.9%	VPA: started in a dosage of 10 mg/kg/day, increments of 5 mg/kg/day, maximum dosage of 20–30 mg/kg/day (19)	
Suo et al. (2021)	China	English	70 randomized, 64 analyzed	LEV 8.47 ± 2.13	LEV 65.6%	LEV: initial dose was set at 10 mg/kg/day, the dose is increased once every 7 days, and is maintained at 20–60 mg/kg/day (32)	Secondary: any adverse events
				OXC 8.62 ± 2.21	OXC 59.4%	OXC: initial dose was set at 10 mg/kg/day, increase the dose to 5–10 mg/kg/day every 5–7 days, and is maintained at 20–46 mg/kg/day (32)	
Tacke et al. (2016)	Germany	English	44 randomized	6–12 for total	NR	LEV: final dosage: 30 mg/kg body weight per day, reduction to 20 mg/kg body weight in case of adverse effects (22)	Primary: seizure freedom rate, treatment withdrawal due to any serious adverse events. Secondary: any adverse events, rate of dropped out
						STM: daily 6 mg/kg body weight with the option of a reduction to 4 mg/kg (22)	
Tacke et al. (2018)	Germany	English	44 randomized	6–12 for total	NR	LEV: final dosage: 30 mg/kg body weight per day, reduction to 20 mg/kg body weight in case of adverse effects (22)	Secondary: the proportion of patients whose EEG to be normalized
						STM: daily 6 mg/kg body weight with the option of a reduction to 4 mg/kg (22)	
Tan et al. (2014)	China	Chinese	108 randomized	6–11 for total	59.3% for total	Not clearly stated	Primary: seizure freedom rate. Secondary: any adverse events, 50% remission rate
Tao and Wang. (2012)	China	Chinese	44 randomized	OXC 7.58 ± 2.17	OXC 52.0%	OXC: started in a dosage of 8–10 mg/kg/day, increments of 5–10 mg/kg/day, maintained dosage of 20–30 mg/kg/day (25)	Primary: seizure freedom rate. Secondary: 50% remission rate, the proportion of patients whose EEG to be normalized, improvement in cognitive function
				VPA 6.61 ± 2.30	VPA 47.4%	VPA: started in a dosage of 10 mg/kg/day, increments of 5–10 mg/kg/day, maintained dosage of 20–30 mg/kg/day (19)	
Yuan 2021	China	Chinese	56 randomized	LTG + VPA 9.53 ± 1.43 (6–13)	LTG + VPA 69.0%, VPA 59.3%	LTG: started in a dosage of 0.15 mg/kg/day, maximum dosage of 5 mg/kg/day (19)	Primary: seizure freedom rate. Secondary: any adverse events, 50% remission rate
				VPA 9.08 ± 1.72 (7–11)		VPA: started in a dosage of 10–15 mg/kg/day, maximum dosage of 20–30 mg/kg/day (19)	
Zhang J et al. (2015)	China	Chinese	160 randomized	OXC 7.4 (3–11)	OXC 56.8%	OXC: started in a dosage of 5–10 mg/kg/day, increments of 5–10 mg/kg/day, maximum dosage of 20–30 mg/kg/day (88)	Primary: seizure freedom rate. Secondary: 50% remission rate, improvement in cognitive function
				CBZ 7.9 (2–12)	CBZ 58.3%	CBZ: started in a dosage of 5–7.5 mg/kg/day, maximum dosage of 20 mg/kg/day (19)	
Zhang et al. (2020)	China	Chinese	60 randomized	LEV 8.20 ± 1.60 (6–12), CBZ 8.15 ± 1.01 (5–12)	LEV 53.3%	LEV: started in a dosage of 10 mg/kg/day, increments of 10 mg/kg/day, maximum dosage of 20 mg/kg/day (30)	Primary: seizure freedom rate. Secondary: any adverse events, improvement in cognitive function
					CBZ 56.7%	CBZ: 10–20 mg/kg/day (30)	
Zhou T (2011)	China	Chinese	69 randomized	(5–14) for total	LEV 60.6%	LEV: started in a dosage of 5–10 mg/kg/day, increments of 5–10 mg/kg/day, maintained dosage of 10–50 mg/kg/day (33)	Primary: seizure freedom rate. Secondary: any adverse events, 50% remission rate
					VPA 66.7%	VPA: started in a dosage of 10 mg/kg/day, increments of 5 mg/kg/day (36)	

CBZ: carbamazepine; CLB: clobazam; CZP: clonazepam; LEV: levetiracetam; LTG: lamotrigine; NR: not reported; OXC: oxcarbazepine; STM: sulthiame; TPM: topiramate; VPA: valproic acid.

### Methodological Quality of the Included Studies

**FIGURE 2:**
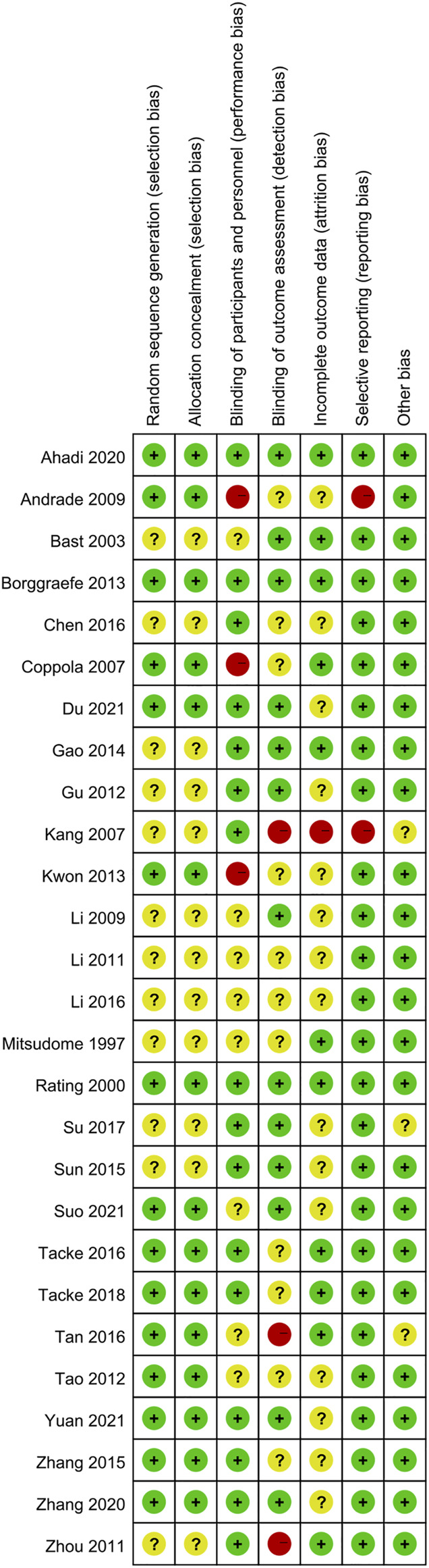
Risk of bias assessment for the 27 included studies.

The results of the risk of bias in the included studies are shown in Figure 2. The risk of bias for random sequence generation in twelve studies (not described) was unclear (12/27, 44.4%). The risk of bias for allocation concealment of twelve included studies was unclear because they did not provide the details of allocation concealment (12/27, 44.4%). In some of the included studies (11/27; 40.7%), blinding of participants and personnel was unspecified or not performed. Three included studies showed a high risk of detection bias because all of their primary and secondary outcomes were recorded by the patients’ parents, which could affect the accuracy of the results. Most of the included studies exhibited an unclear risk of bias for incomplete outcome data (15/27, 55.6%). Almost all included studies were at low risk of selective reporting (25/27, 92.6%), while two studies showed a high risk of selective reporting.

### Outcomes

Summary of the meta-analysis results are showed in Table 2.

**TABLE 2:** Summary of the meta-analysis results.

Outcomes	Effect Size Summary (RR/WMD)	95% CI	Z	*p*-value for Z	Heterogeneity		
					Heterogeneity statistic	I-squared (%)	*p*
Primary outcomes	—	—	—	—	—	—	—
seizure freedom rates	1.25	0.52, 2.99	0.50	0.616	9.99	90.0	0.002
LEV-CBZ	0.90	0.75, 1.06	1.27	0.205	0.00	0.0	0.951
LEV-STM	1.18	0.92, 1.50	1.31	0.191	0.43	0.0	0.512
LEV-OXC	1.20	0.94, 1.53	1.46	0.145	2.05	2.2	0.360
LEV-VPA	1.14	0.96, 1.36	1.45	0.147	0.02	0.0	0.989
OXC-VPA	—	—	—	—	—	—	—
treatment withdrawal (Due to serious adverse events)	5.12	1.19, 22.01	2.19	0.028	0.00	0.0	0.975
LEV-STM							
Secondary outcomes	—	—	—	—	—	—	—
any adverse events	0.62	0.25, 1.59	0.99	0.324	0.38	0.0	0.539
LEV-CBZ	5.12	1.19, 22.01	2.19	0.028	0.00	0.0	0.975
LEV-STM	0.57	0.19, 1.73	0.99	0.321	4.77	58.0	0.092
LEV-OXC	0.64	0.22, 1.81	0.85	0.397	6.03	66.9	0.049
LEV-VPA	—	—	—	—	—	—	—
Rate of dropped out LEV-STM	3.71	1.36, 10.11	2.56	0.010	0.14	0.0	0.705
50% remission rate	0.96	0.57, 1.61	0.17	0.866	0.06	0.0	0.970
OXC-VPA	0.61	0.31, 1.20	1.44	0.151	1.54	0.0	0.463
LEV-VPA	—	—	—	—	—	—	—
EEG normalized	4.61	2.12, 10.01	3.87	0.000	0.03	0.0	0.872
STM-Placebo	1.27	0.94, 1.71	1.53	0.125	0.89	0.0	0.344
LEV-CBZ	0.82	0.42, 1.61	0.58	0.565	0.35	0.0	0.555
CBZ-VPA	1.33	0.66, 2.69	0.80	0.424	0.00	0.0	0.997
OXC-VPA	—	—	—	—	—	—	—
cognitive ability (CBZ-LEV)	−4.74	−9.17, −0.30	2.09	0.036	40.12	95.0	0.000
VIQ	−1.37	−3.86, 1.12	1.08	0.280	5.14	61.1	0.076
PIQ	−1.80	−2.55, -1.05	4.70	0.000	1.26	0.0	0.531
FIQ	—	—	—	—	—	—	—

CBZ: carbamazepine; FIQ: full-scale intelligence quotient; LEV: levetiracetam; OXC: oxcarbazepine; PIQ: performance intelligence quotient; STM: sulthiame; VIQ: verbal intelligence quotient VPA: valproic acid.

#### 3.1.1 Primary Outcomes

**FIGURE 3:**
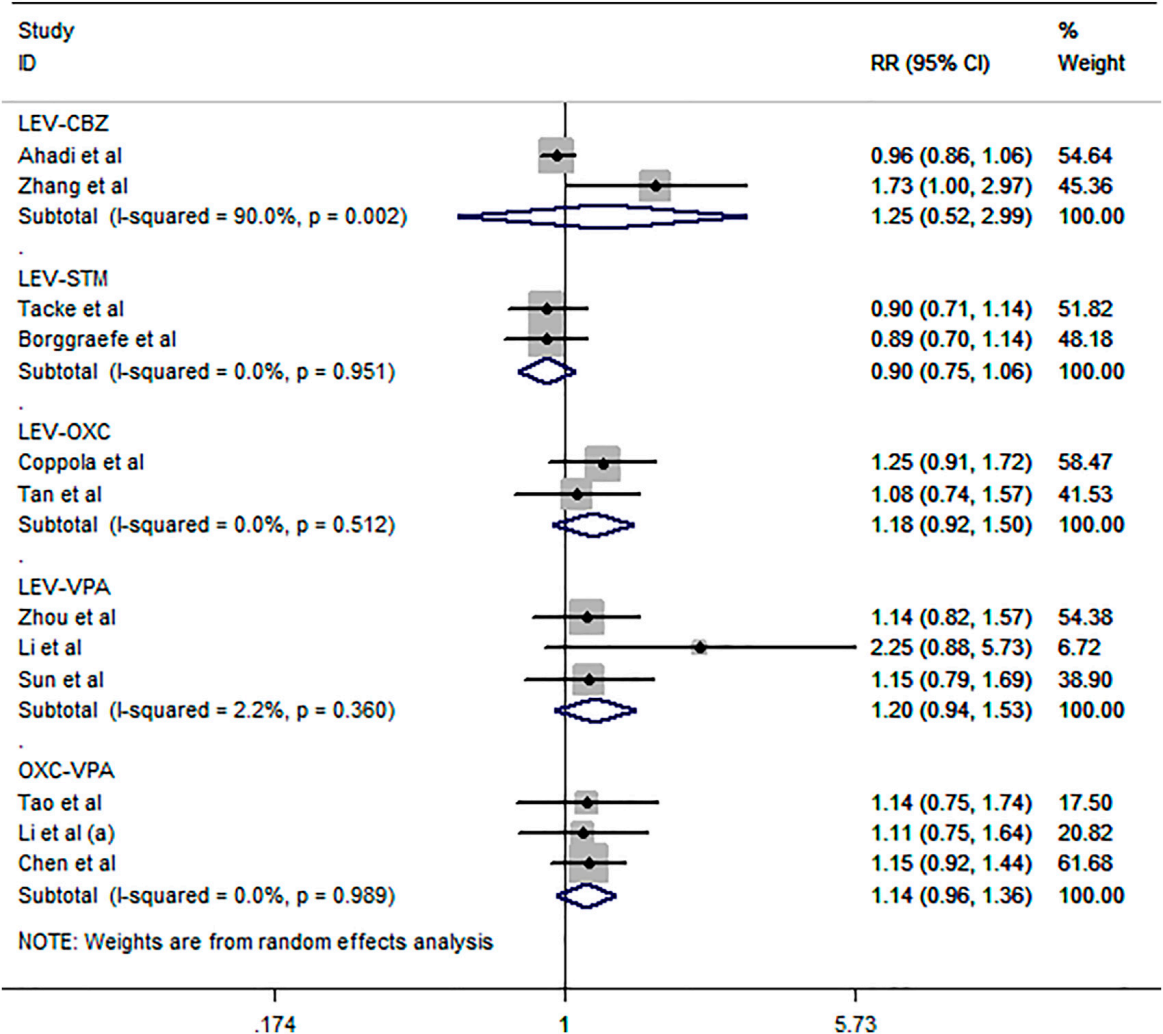
Forest plot of RRs and 95% CIs for seizure freedom rates.

Twelve studies (Coppola, et al., 2007; Li ZH and Huang, 2011; Zhou T, 2011; Tao and Wang, 2012; Borggraefe, et al., 2013; Sun, 2015; Chen CY and Zheng, 2016; JY, 2016; Li J, 2016; Tacke, et al., 2016; Ahadi, et al., 2020; Zhang et al., 2020) involving 790 participants were included in the meta-analysis to explore the seizure freedom rates (complete control) of ASM treatment in BECTS. The results of the pooled analysis indicated that LEV did not demonstrate a higher probability of seizure freedom than CBZ [RR = 1.25, 95% CI (0.52, 2.99)]. However, this comparison showed notable heterogeneity (*I* ^*2*^ = 90.0%), which is difficult to eliminate and, therefore, excluded from our meta-analysis. In addition, there was no significant difference between LEV and STM, OXC and VPA for the probability of seizure freedom rates [RR = 0.90, 95% CI (0.75, 1.06), *I* ^*2*^ = 0.0%; RR = 1.18, 95% CI (0.92, 1.50), *I* ^*2*^ = 0.0%; RR = 1.20, 95% CI (0.94, 1.53), *I* ^*2*^ = 2.2%] (Figure 3). Additionally, there was inconclusive evidence of whether OXC was associated with a higher probability of seizure freedom than VPA [RR = 1.14, 95% CI (0.96, 0.36), *I* ^*2*^ = 0.0%].

**FIGURE 4:**
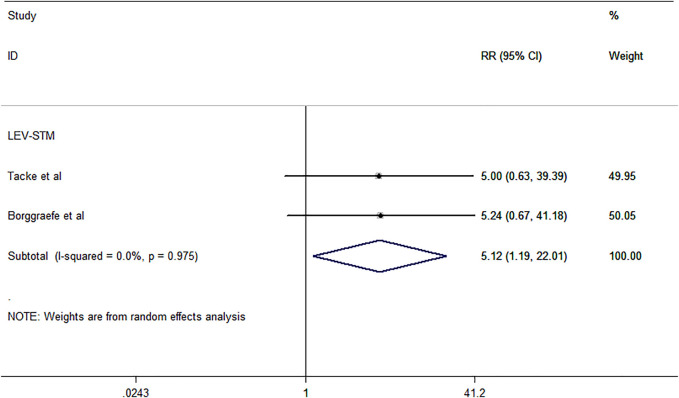
Forest plot of RRs and 95% CIs for the proportion of treatment withdrawal due to serious adverse events.

The outcome of treatment withdrawal due to serious adverse events was assessed in two studies (Borggraefe, et al., 2013; Tacke, et al., 2016) included in this meta-analysis. The pooled analysis data showed that LEV revealed a higher probability of treatment discontinuation or change due to adverse events than STM [RR = 5.12, 95% CI (1.19, 22.01), *I* ^*2*^ = 0.0%] (Figure 4). Additional studies that were not included in the meta-analysis are shown in Table 1.

#### 3.1.2 Secondary Outcomes

**FIGURE 5:**
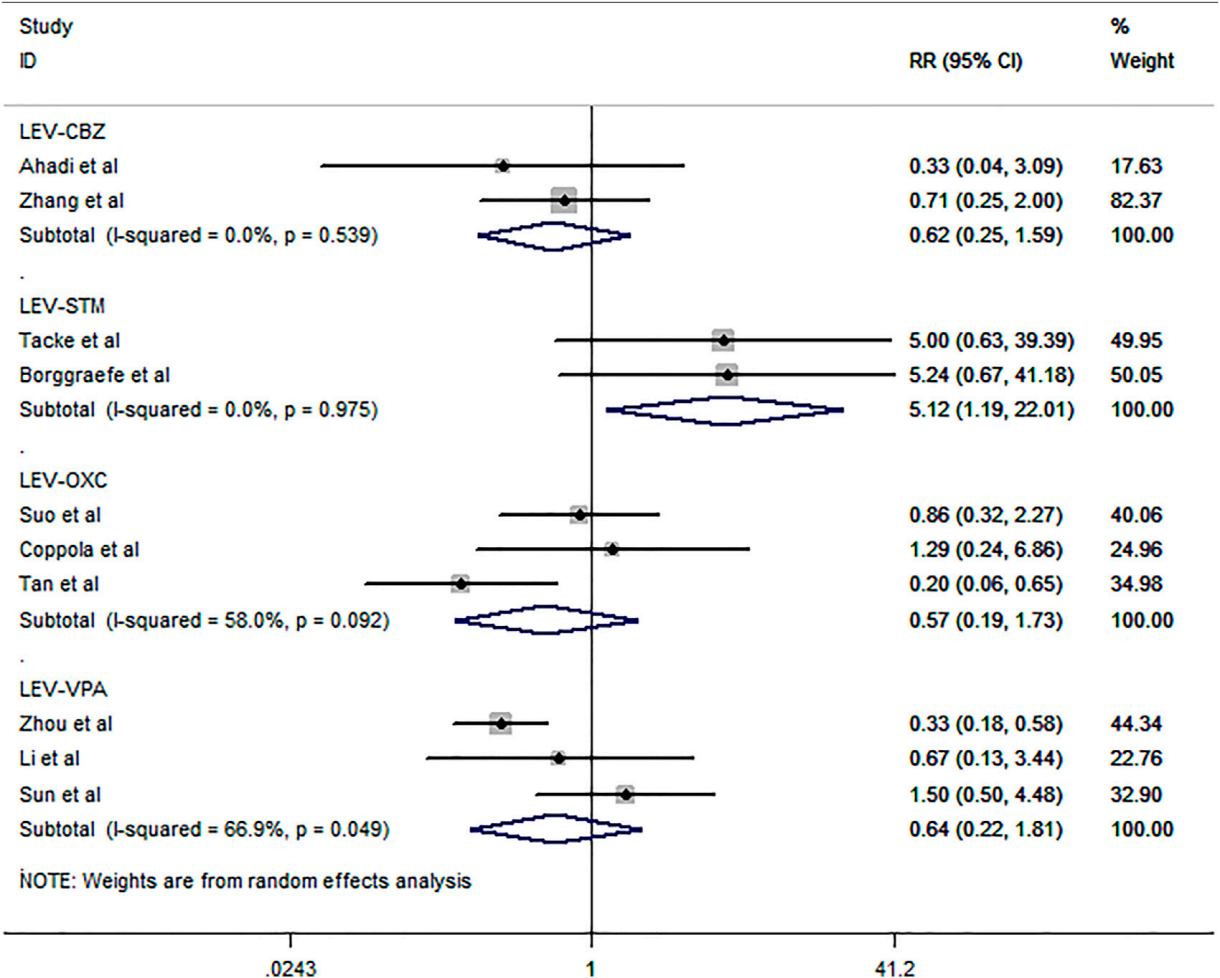
Forest plot of RRs and 95% CIs for rate of any adverse events.

**FIGURE 6:**
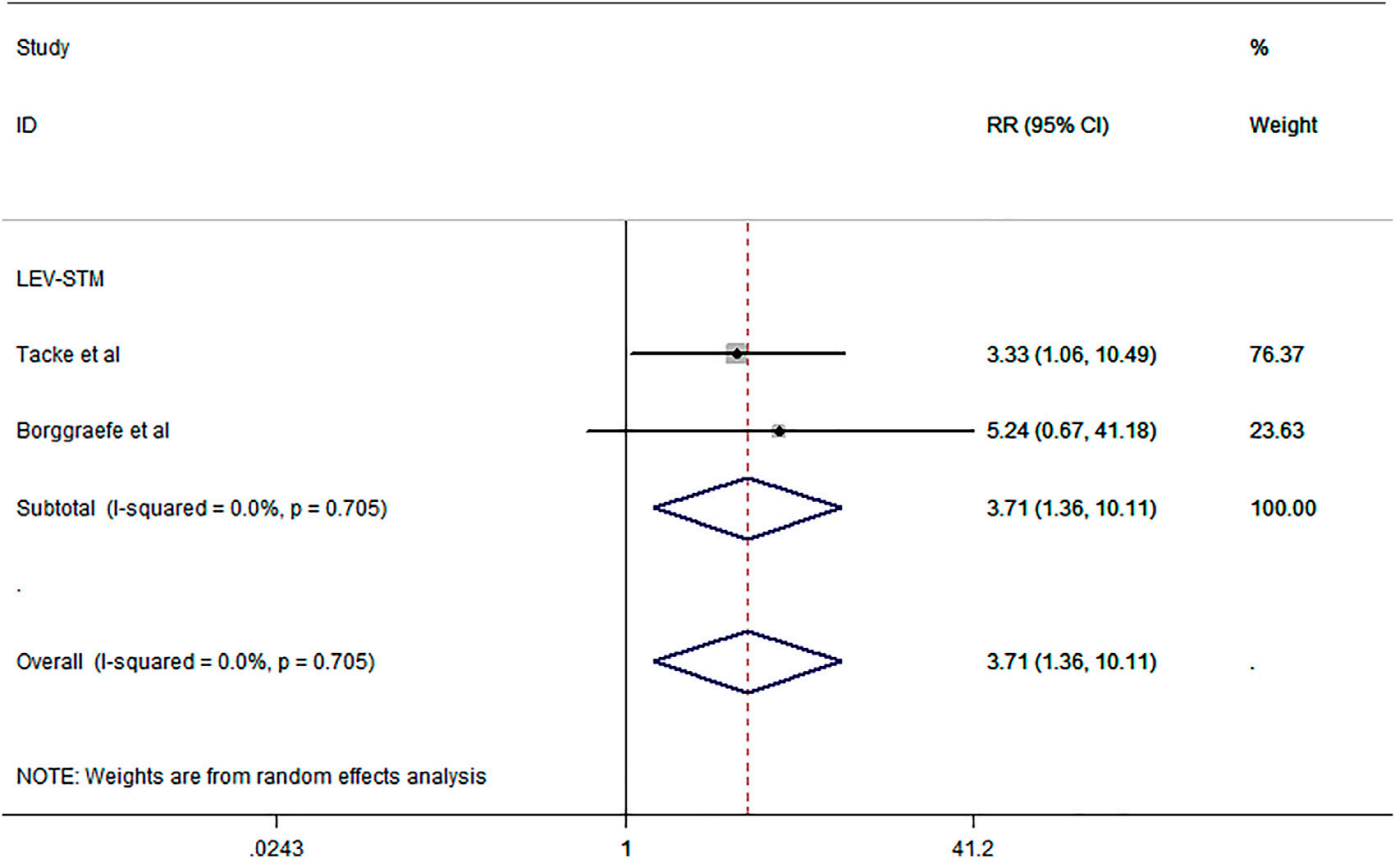
Forest plot of RRs and 95% CIs for rate of dropped out.

Ten studies (Coppola, et al., 2007; Li ZH and Huang, 2011; Zhou T, 2011; Sun, 2015; Borggraefe, et al., 2013; JY, 2016; Tacke, et al., 2016; Ahadi, et al., 2020; Zhang et al., 2020; Suo, et al., 2021) including 589 subjects were included in the meta-analysis to investigate the proportion of patients who experienced any adverse events (total rate of ASMs) of antiepileptic drugs in the BECTS (Figure 5). The pooled analysis results showed that the probability of experiencing any adverse events between LEV and OXC and VPA was not significantly different [RR = 0.57, 95% CI (0.19, 1.73); RR = 0.64, 95% CI (0.22, 1.81)], with moderate heterogeneity (*I* ^*2*^ = 58.0%; *I* ^*2*^ = 66.9%). Additionally, there was no evidence that LEV and CBZ were associated with a different risk of experiencing adverse events [RR = 0.62, 95% CI (0.25, 1.59), *I* ^*2*^ = 0.0%]. LEV, however, was more likely to result in more adverse events than STM [RR = 5.12, 95% CI (1.19, 22.01), *I* ^*2*^ = 0.0%]. Two studies (Borggraefe, et al., 2013; Tacke, et al., 2016) involving 87 subjects were included in the meta-analysis to investigate the proportion of patients who dropped out for any reason. The pooled analysis results showed that LEV demonstrated a higher probability of dropping out for any reason [RR = 3.17, 95% CI (1.36, 10.11), *I* ^*2*^ = 0.0%] (Figure 6).

**FIGURE 7:**
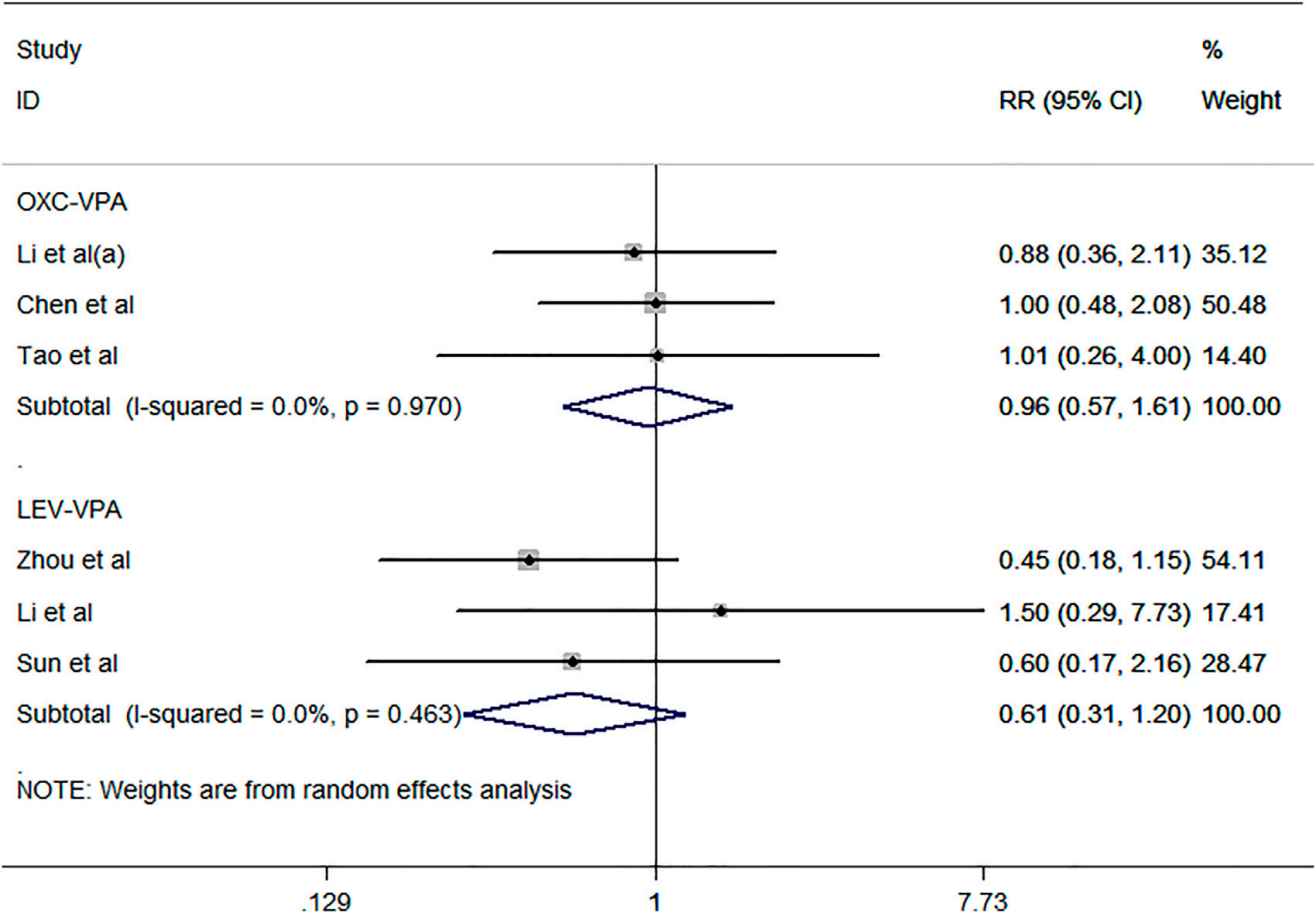
Forest plot of RRs and 95% CIs for 50% remission rate.

**FIGURE 8:**
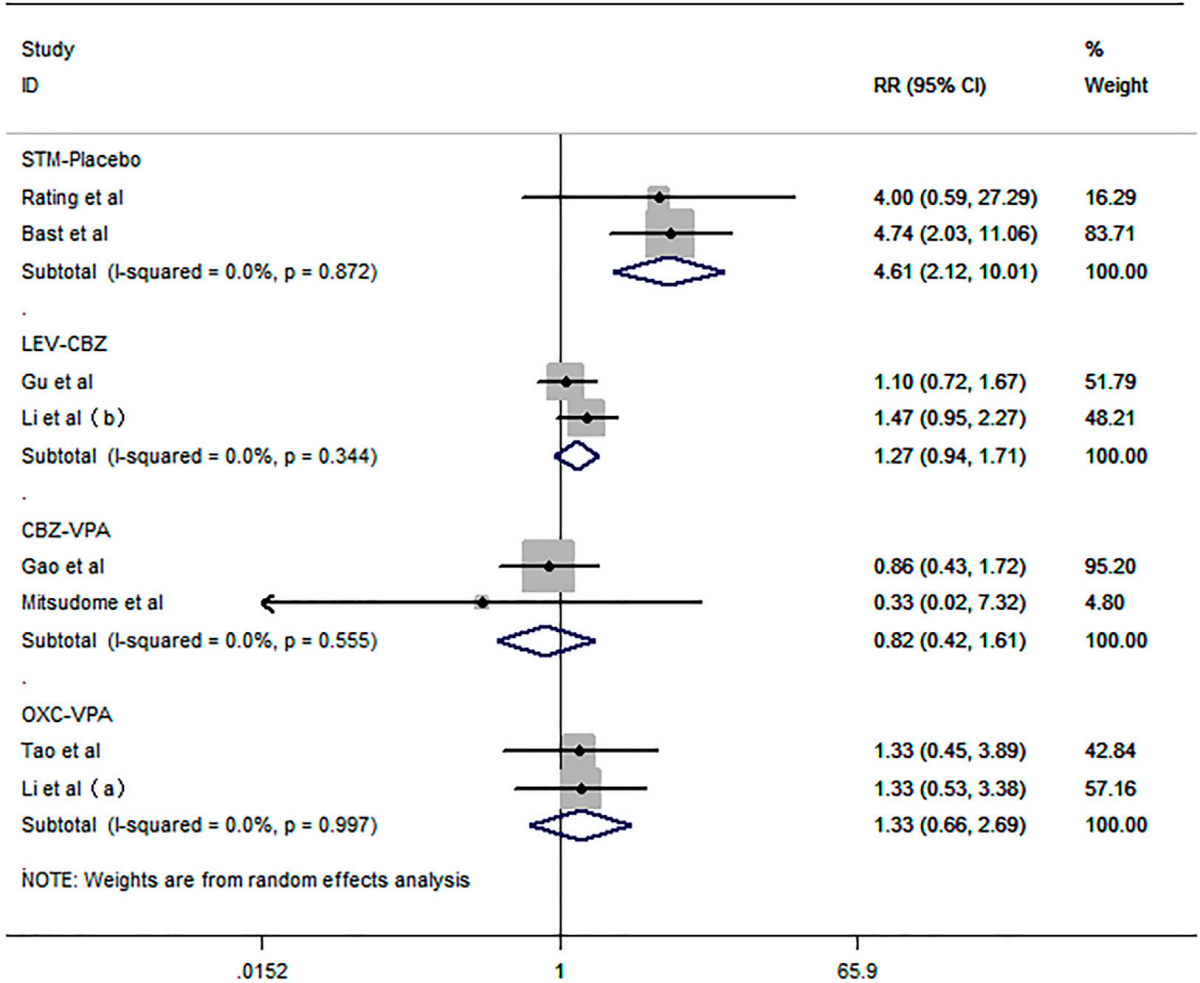
Forest plot of RRs and 95% CIs for the rate of EEG normalization.

Six studies (Li ZH and Huang, 2011; Zhou T, 2011; Tao and Wang, 2012; Sun, 2015; Chen CY and Zheng, 2016; Li J, 2016) including 391 subjects were included in the meta-analysis to investigate the 50% remission rate after antiepileptic drug treatment in BECTS. As revealed by the pooled analysis data (Figure 7), no differences were noted between VPA and OXC or LEV for the proportion of patients with a reduction in seizure frequency of more than 50% compared to baseline [RR = 0.96, 95% CI (0.57, 1.61), *I* ^*2*^ = 0.0%; RR = 0.61, 95% CI (0.31, 1.20), *I* ^*2*^ = 0.0%]. The rate of EEG normalization after treatment was evaluated in eight studies (Mitsudome, et al., 1997; Rating, et al., 2000; Bast, et al., 2003; Gu HF, 2012; Tao and Wang, 2012; CH, 2014; Li J, 2016) included in this meta-analysis (Figure 8). As the pooled analysis data showed, STM was more likely to result in EEG normalization than placebo [RR = 4.61, 95% CI (2.12, 10.01), *I* ^*2*^ = 37.9%]. VPA did not indicate a higher probability of leading to EEG normalization compared to CBZ and OXC [RR = 0.82, 95% CI (0.42, 1.61), *I* ^*2*^ = 37.9%; RR = 1.33, 95% CI (0.66, 2.69), *I* ^*2*^ = 0.0%]. There were no differences in the probability of participant withdrawal when comparing LEV versus CBZ [RR = 1.27, 95% CI (0.94, 1.71), *I* ^*2*^ = 37.9%].

**FIGURE 9:**
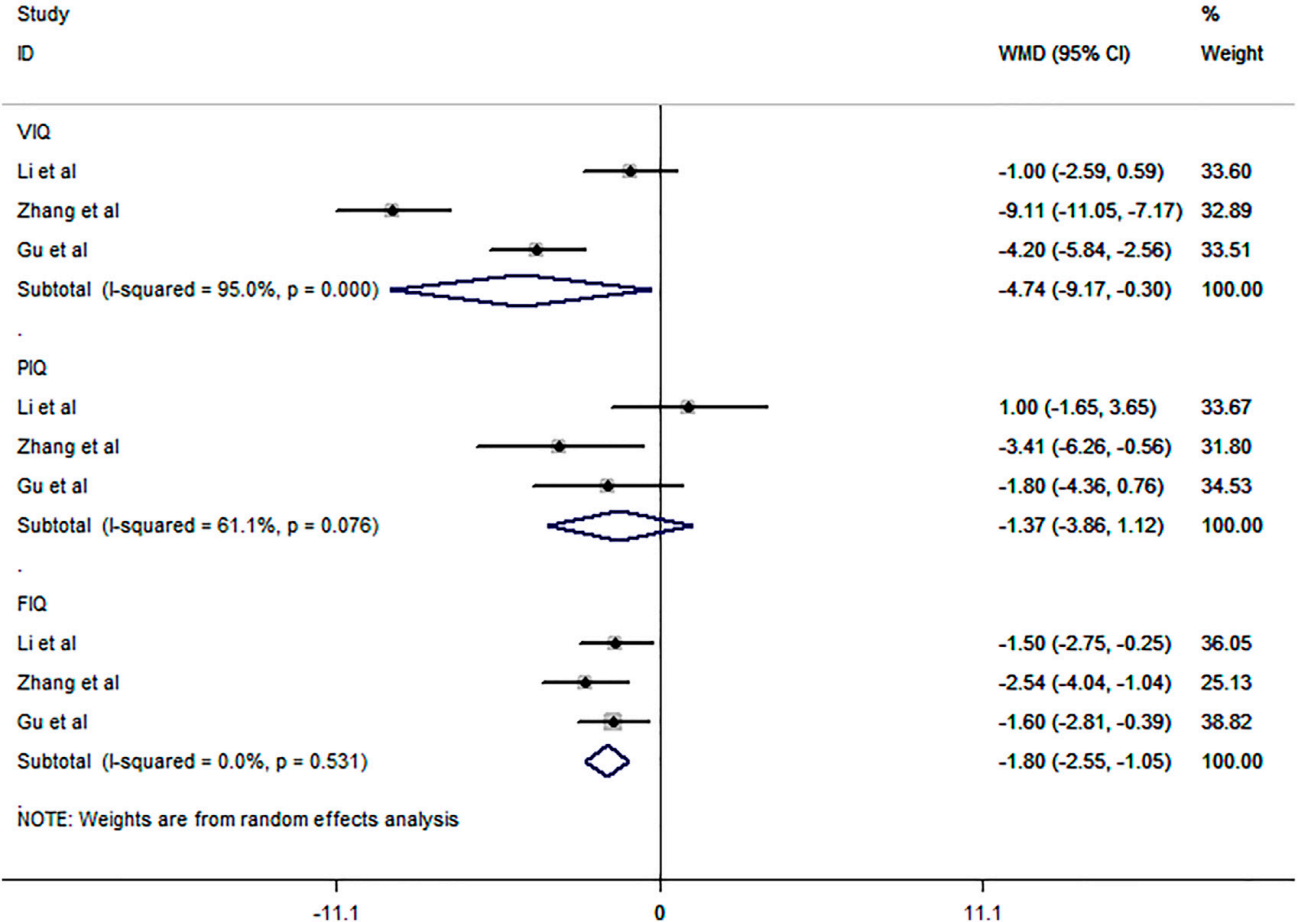
Forest plot of WMDs and 95% CIs for proportion of patients with improved cognitive function (LEV vs. CBZ).

Three studies (Li SM et al., 2009; Gu HF, 2012; Zhang et al., 2020) involving 220 subjects were included in the meta-analysis for the proportion of patients with improved cognitive function. The three eligible studies all compared CBZ and LEV, as shown by the pooled analysis (Figure 9). LEV was more likely to improve the VIQ and FIQ in BECTS than CBZ [RR = −4.74, 95% CI (−9.17, −0.30), *I* ^*2*^ = 95.0%; RR = −1.80, 95% CI (−2.55, −1.05), *I* ^*2*^ = 0.0%]. As described in the primary outcomes section, we excluded the comparison of LEV and CBZ on VIQ due to its notable heterogeneity (*I* ^*2*^ = 95.0%), which is difficult to remove. In addition, there were no differences in the comparison of LEV and CBZ on PIQ [RR = -1.37, 95% CI (−3.86, 1.12), *I* ^*2*^ = 61.1%].

### Publication Bias

Publication bias in this review did not be estimated because of inadequate inclusive studies.

## 4 Discussion

The main results of this systematic review and meta-analysis are as follows: 1) treatment with LEV in patients with BECTS did not significantly reduce the seizure freedom rate when compared with STM, OXC, and VPA, as well as when comparing OXC versus VPA; 2) LEV can significantly increase the probability of treatment withdrawal due to serious adverse events and dropping out for any reason than STM; 3) LEV did not affect the proportion of any adverse events when compared with CBZ, OXC, and VPA; however, it may significantly improve cognitive performance relative to CBZ; 4) There were no differences in the probability of 50% remission rate when comparing VPA versus LEV and OXC; 5) STM was related to a higher probability of EEG to be normalized, while it was no different between VPA versus CBZ, OXC, and LEV versus CBZ.

To our knowledge, our work complements recent studies on the efficacy and tolerability of ASMs in patients with BECTS and enables, for the first time, a comprehensive assessment of the comparative efficacy and tolerability of ASMs without language restrictions. In this study, the seizure freedom rates, 50% remission rate, proportion of EEG to be normalized, and cognition improvement were considered outcomes of efficacy, whereas the proportion of treatment withdrawal due to serious adverse events, rate of any adverse events, and rate of dropout were regarded as tolerability. Ultimately, we identified 27 RCTs involving 1737 subjects evaluating 9 ASMs, 19 of which included 1,224 subjects for meta-analysis.

First, seizure freedom rates are often used to assess the effectiveness of ASMs in patients with BECTS (“Considerations on designing clinical trials to evaluate the place of new antiepileptic drugs in the treatment of newly diagnosed and chronic patients with epilepsy,” 1998). However, our meta-analysis failed to demonstrate a significant difference in the probability of seizure freedom rates for ASM comparisons. As shown in Table 2, LEV was not associated with a higher probability of seizure freedom rates than STM, OXC, or VPA, and there was no difference in the seizure freedom rate between OXC and VPA. Similar results were found in the single studies identified by this systematic review that were not included in the meta-analysis (fewer than two studies were available). Kang et al. evaluated the effect of TPM and CBZ using efficacious doses in BECTS and reported that the percentage of seizure-free patients did not differ between TPM and CBZ (Kang, et al., 2007). A study (Kwon, et al., 2013) conducted by Kwon et al. also reported that OXC monotherapy was ineffective in seizure freedom for children with BECTS. Compared to CBZ, CLB and OXC did not significantly reduce the rate of seizure freedom (Andrade, et al., 2009; Zhang J et al., 2015). In addition, a comparative study of ASM efficacy also demonstrated that CBZ was not related to a higher probability of seizure freedom than VPA (CH, 2014). All of the above results seem to indicate that there is no significant difference in the effect of different ASMs on the seizure remission of BECTS. Furthermore, single studies provide some evidence for the effectiveness of ASM treatment in patients with BECTS compared with placebo. Rating et al. evaluated the efficacy of STM as monotherapy in BECTS and reported that STM significantly increased the seizure freedom rates compared with placebo (Rating, et al., 2000). LTG combined with VPA significantly increased the rate of seizure freedom in patients with BECTS compared with VPA monotherapy (LY, 2021). Evidence of the effectiveness of ASMs in the seizure freedom rates of BECTS mainly comes from studies comparing with the placebo group, while studies comparing the efficacy of difference ASMs showed no difference, which may indicate that the use of ASMs in BECTS patients can effectively control seizures, whereas the curative effect between different ASMs was equivalent.

Patients in BECTS with a reduction in seizure frequency of more than 50% compared to baseline after drug treatment would be considered effective (“Considerations on designing clinical trials to evaluate the place of new antiepileptic drugs in the treatment of newly diagnosed and chronic patients with epilepsy,” 1998; Kwon, et al., 2013; Zhang J et al., 2015); however, as with seizure freedom rates, we similarly found no difference in the rate of 50% remission when comparing VPA with OXC and LEV in this meta-analysis. This result was supported by the single studies we included. In the present study, all single comparative studies involving ASMs (OXC, CBZ, LEV, VPA, and LTG) demonstrated no difference in the 50% remission rate between ASMs, even compared with placebo. Moreover, although both the seizure freedom rate and 50% remission rate were commonly used to evaluate the efficacy of ASMs, the results of these two outcomes might be different in the same comparative study. This differentiation was supported by a study (LY, 2021) conducted by Yuan et al. Data from this study revealed a significant difference in the seizure freedom rate but not the 50% remission rate when comparing LTG with placebo.

There has been a long debate about whether ASM use might promote EEG changes in patients with BECTS (Tenney et al., 2016; Kim, et al., 2018; Tacke, et al., 2018; Han et al., 2020). A retrospective study (Kim, et al., 2018) compared the EEG of BECTS with matched onset age in the two groups and found no difference in the abnormal EEG duration between treated with ASMs and untreated children, which indicated that ASM treatment did not shorten the duration of EEG abnormalities. Another retrospective study (Han, et al., 2020) also found that treatment with OXC in BECTS did not affect the pattern of spike disappearance. However, other studies (Kanemura, et al., 2018; Tacke, et al., 2018) demonstrated that ASM treatment in BECTS might have a positive effect on EEG. For example, Tacke et al. conducted an RCT study (Tacke, et al., 2018) to examine the influence of ASM treatment (STM and LEV) on EEG and revealed that treatment with both STM and LEV significantly reduced the spike-wave index, with no differences between the two treatment groups. A study (Kanemura, et al., 2018) conducted by Kanemura et al. reported similar results and revealed that LEV seems superior to CBZ and VPA in its ability to suppress rolandic discharges (RDs) interictally in children with BECTS. Our meta-analysis results showed that STM was related to a higher probability of EEG normalization than placebo, while there was no difference between VPA versus CBZ, OXC, and LEV versus CBZ. This analysis was consistent with previous partial studies and supported that ASM (STM) treatment could effectively suppress the interictal discharge of BECTS patients, but there was no difference in the comparative effect of different ASMs.

In addition, we noted that LEV could significantly improve cognitive performance relative to CBZ, which provided some evidence for the effectiveness of ASMs in improving cognitive function. As shown in this meta-analysis, LEV was associated with improvement of full-scale intelligence quotient (FIQ) but not performance intelligence quotient (PIQ) when compared with CBZ. This result was partially supported by the single RCT study (NS, 2017) that we included. The study (NS, 2017) conducted by Su et al. reported that compared with VPA monotherapy, LEV combined with VPA could significantly improve cognitive ability. Some clinical studies (Han and Kim, 2018; Operto et al., 2019) also supported the effectiveness of ASMs in cognitive function. A 2-years follow-up study (Operto, et al., 2019) of LEV monotherapy revealed significant improvements in verbal comprehension, perceptual reasoning, working memory, and processing speed, suggesting that LEV has a protective effect on cognitive function. A retrospective study (Han & Kim, 2018) also reported improvements in language and problem-solving performance in children with BECTS were greater for LTG and OXC than for TPM. However, except for one single study (Tao and Wang, 2012) (VPA versus OXC, Tao and Wang. 2012), all comparative RCT studies (Zhang J et al., 2015; NS, 2017) included in this systematic review revealed no difference in the improvement of cognitive ability between ASMs. Compared with placebo, OXC monotherapy was not effective for the improvement of cognitive ability in BECTS (Kwon, et al., 2013). This result may suggest that the most commonly used ASMs in the clinic have no significant difference in the improvement of cognitive ability in BECTS patients, while whether some controversial ASMs (such as OXC and VPA) can improve cognitive ability still needs to be further explored by blank control studies.

Although a number of clinical studies have reported the occurrence of adverse reactions after using ASMs in BECTS, such as weight gain or loss, sleep disorder, fatigue, erythema, and loss of appetite (Coppola, et al., 2007; Borggraefe, et al., 2013; Ahadi, et al., 2020), the difference in the proportion and severity of adverse reactions caused by different ASMs has not been well clarified. Our results provided some evidence for this issue. On the one hand, LEV can significantly increase the probability of treatment withdrawal due to serious adverse events compared to STM; on the other hand, LEV was associated with a higher probability of any adverse events than STM but not clearly when compared to CBZ, OXC, and VPA. Additionally, there were no differences in the probability of dropping out when comparing LEV versus STM. The above results of the meta-analysis showed that LEV versus STM was related to a higher probability of treatment withdrawal due to serious adverse events, experiencing any adverse events, and dropping out for any reason, suggesting that STM was superior to LEV in terms of tolerability. No difference in the proportion of any adverse events between LEV and other ASMs might indicate that STM was also better tolerated than other ASMs; however, no RCT studies have been performed to evaluate the proportion of any adverse events between STM and other ASMs (other than LEV); hence, there was inconclusive evidence about tolerability on STM versus other ASMs. In addition, one single study (Rating, et al., 2000) conducted by Rating et al. reported that STM was relevant to a lower probability of dropping out than placebo and suggested a high retention rate with STM. The remaining single studies (Kang, et al., 2007; Andrade, et al., 2009; Li J, 2016; Du ZZ et al., 2021; LY, 2021) included in this paper involving TPM, CBZ, CLB, OXC, VPA, and LTG revealed no difference in the proportion of treatment withdrawal due to serious adverse events, experiencing any adverse events, and dropping out between ASMs.

There are some limitations to this study. First, although this study collected and analyzed literature without language limitations, more than half of the studies included were conducted in China, and regional differences may impact the results of studies. Second, most outcomes, such as any adverse events, dropped out for any reason, and normalized EEG was measured in only two studies, leading to imprecision in the outcomes. Third, considering the lack of literature and relevant data, we did not conduct subgroup analyses of the dosage of ASM in this study. Fourth, due to insufficient studies for each outcome comparison, we did not formally assess publication bias.

## 5 Conclusion

The present study indicated that STM could reduce interictal EEG activity in patients with BECTS, and it is better tolerated than LEV in patients with BECTS, while it revealed no difference in efficacy. LEV demonstrated comparable safety to CBZ but better cognitive improvement. LEV is not superior to VPA in efficacy and tolerability. In addition, OXC was not better at seizure control and EEG normalization than VPA. Overall, this study indicated some discrepancies in efficacy and tolerability among ASMs used in patients with BECTS, and more RCT studies are required to examine the efficacy and tolerability of different ASMs to ascertain the optimum antiepileptic drug treatment.

## Data Availability

The original contributions presented in the study are included in the article/Supplementary Material, further inquiries can be directed to the corresponding authors.
